# The usefulness of the intergluteal cleft as a possible anatomical landmark of the neuraxial midline in obstetrics: a prospective observational study

**DOI:** 10.1097/EJA.0000000000002416

**Published:** 2026-05-07

**Authors:** Suji Pararajasingam, Maame Aduse-Poku, Rajeev Jeevananthan, Gillian Radcliffe, Marwa Salman, Neel Desai

**Affiliations:** From the Department of Anaesthesia, Guy's and St Thomas’ NHS Foundation Trust, London, UK (SP, MAP, RJ, GR, MS, ND)

Editor,

Neuraxial blockade is an essential method of providing analgesia and anaesthesia in obstetrics. Conventionally, ascertaining the correct point of needle insertion to conduct a neuraxial block has relied upon the palpation of landmarks, namely the iliac crests and spinous processes, to identify the intercristal line and midline of the spine. The obstetric population has risk factors, including a more pronounced lumbar lordosis and the presence of soft tissue oedema, that can lead to significant difficulties in the recognition of neuraxial landmarks by palpation alone.^1^ Neuraxial placement has been associated with cauda equina syndrome, pararesis and paraplegia, and these epidural and spinal procedures were the most commonly used regional analgesic or anaesthetic techniques in a closed claims analysis.^2^ Preprocedural ultrasound guidance was demonstrated in a previous meta-analysis and systematic review to increase the first pass success rate,^3^ and is recommended by the European Society of Anaesthesiology and Intensive Care.^4^

Not all health care professionals, however, are competent and experienced in the use of ultrasound, and not all institutions have ultrasound machines. Given this, in the absence of neuraxial ultrasound, the superior aspect of the intergluteal cleft (the midline groove extending from the sacrum to the anal verge, separating the left and right gluteal regions) could be a supplementary landmark that the anaesthetist may use to identify the neuraxial midline in obstetrics. Of relevance, the sacral hiatus, which represents the incomplete midline fusion of the posterior elements of the fifth and sometimes fourth sacral vertebra, may be palpated through the intergluteal cleft.^5^ In view of this relationship between the intergluteal cleft and sacral hiatus, we postulated that the intergluteal cleft might be an indicative marker of the neuraxial midline.

To determine the usefulness of the intergluteal cleft, we designed a prospective observational study to investigate if the intergluteal cleft, when compared with ultrasound as the reference standard, could be used as a supplementary landmark to identify the neuraxial midline in the obstetric patient. Ethical approval for this study (Ethical Committee HSC REC A 23/NI/0116) was provided by the Health and Social Care Research Ethical Committee A of the United Kingdom (Chairperson Dr Mary Murphy) on the 1st September 2023 (IRAS number 291462) and registered with the Clinicaltrials.gov (NCT05983029). We calculated the sample size to be 99 participants based on a population standard deviation of 20 mm in a previous unpublished study and to provide a power of 80% at the significance level of 5%.

The study was conducted in our tertiary obstetric unit at St Thomas’ Hospital, UK between December 2023 and November 2024. Eligible study participants were pregnant patients who were 18 years or older and with a gestational age of 37 weeks or more. They were not eligible for inclusion if they had a past medical history of scoliosis or previous spinal surgery. To obtain a representative sample, we aimed for 50% of participants to have a BMI of at least 30 kg m^−2^. We collected baseline demographic and clinical data, such as age, weight, height, BMI, ethnicity and the presence of hypertensive comorbidities.

**Fig. 1:**
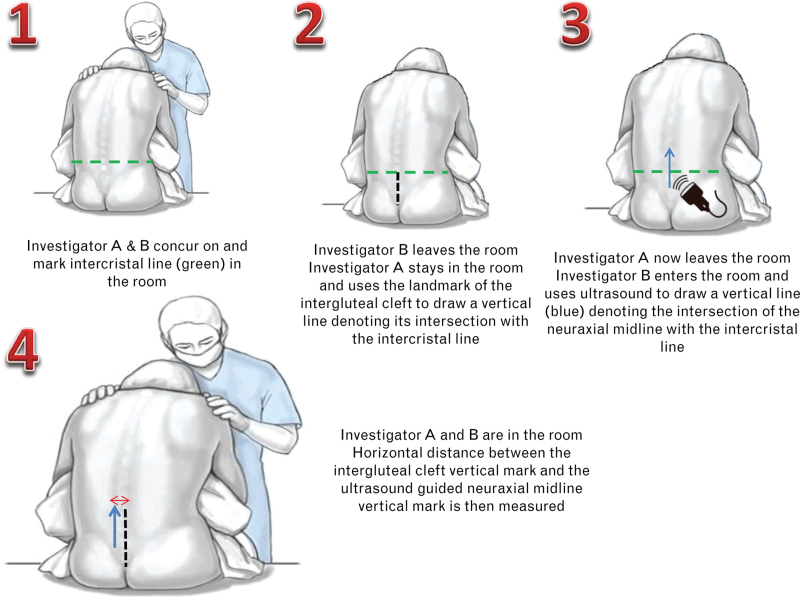
Series of steps followed by the investigators to measure the horizontal distance between the intergluteal cleft and neuraxial midline.

To measure the horizontal distance between the intergluteal cleft and neuraxial midline with the participant in the sitting position, the following steps were performed in a systematic manner (Fig. 1). First, the two investigators, investigator A (SP) and investigator B (ND or MS), both of whom were anaesthetists, palpated the iliac crests and used a spirit level ruler (DK Tools, West Drayton, UK) to adjust the position of the participant to ensure the pelvis was level. Subsequently, with an ink pen, they drew a visible horizontal intercristal line on the back of the participant. Investigator B then left the room. Second, using the spirit ruler and an invisible ultraviolet marker pen, investigator A denoted the intersection of the vertical line from the superior aspect of the intergluteal cleft with the horizontal intercristal line. The spirit ruler served to ensure that the vertical mark was plumb with the superior aspect of the intergluteal cleft. Investigator A subsequently left and investigator B returned to the room. Third, investigator B made a visible vertical mark with an ink pen at the intersection of the neuraxial midline, as established using the middle of the hyperechoic spinous process on the transverse spinous process view on ultrasound with the curved array transducer of the VScan Air (GE HealthCare, Chalfont St Giles, UK), and the horizontal intercristal line. Investigator A then returned to the room. Fourth, now that the two vertical marks had been made at the level of the intercristal line, the two investigators used an ultraviolent light (Mark S G Enterprises, Surrey, UK) to uncover the invisible one. The horizontal distance between these two vertical marks, or the intergluteal cleft and neuraxial midline as determined by ultrasound, was measured in mm by an electronic calliper (RS Components, Northamptonshire, UK).

Data were analysed using SPSS (Version 28.0, IBM, 2023, New York, USA) and tested for normality with the Kolmogorov–Smirnov test. If the data followed a normal distribution they were expressed as the mean ± SD and if the data followed a non-normal distribution they were expressed as the median [IQR]. The effect of ethnicity on the primary outcome was investigated with the ANOVA test if normal or the Kruskal–Wallis *H* test if non-normal in distribution, and the influence of BMI, chronic hypertension, pregnancy induced hypertension and pre-eclampsia were studied with the unpaired t-tests if normal or the Mann–Whitney *U* test if non-normal in distribution.

In all, 100 participants with a mean age of 34 ± 5 years were recruited. BMI at the time of booking was 30.1 [23.1 to 35.2] kg m^−2^, BMI at the time of the study was 34.1 ± 6.6 kg m^−2^ and gestation at the time of the study was 38.4 [37.4 to 39.1] weeks. The numbers (%) were chronic hypertension 1 (1), pregnancy induced hypertension 8 (8), and pre-eclampsia 3 (3). Fifty-two percent of the participants had a BMI ≥30 kg m^−2^ at the time of booking their pregnancy and 72% had a BMI ≥30 kg m^−2^ at the time of recruitment to the study.

**Fig. 2:**
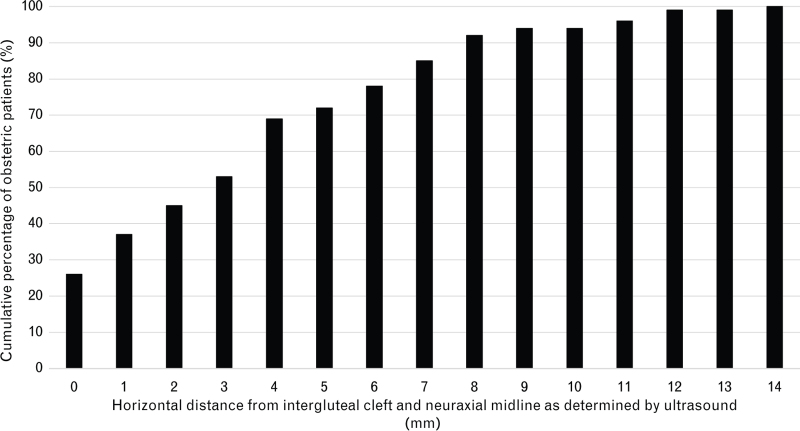
Illustration of the relationship between the horizontal distance from the intergluteal cleft to the neuraxial midline, as determined by ultrasound, and the cumulative percentage of obstetric patients.

The median [IQR] horizontal distance between the vertical lines of the intergluteal cleft and neuraxial midline marked on the intercristal line was 3 [0.2 to 6] mm with a range of 0 mm to 13.9 mm. The median horizontal distance was ≤1 mm in 30% of participants, ≤2 mm in 39%, and ≤4.5 mm in 70% (Fig. 2). In 26% of women, the two vertical lines were superimposed, but in 47% the line from the midpoint near the top of the intergluteal cleft was to the right of the neuraxial midline as determined by ultrasound, and in 27% it was to the left. None of the following factors affected the horizontal distance between the intergluteal cleft and neuraxial midline: ethnicity (*P* = 0.05), BMI at the time of booking the pregnancy (*P* = 0.58) or the BMI at the time of the study (*P* = 0.47).

Importantly, the conventional landmark palpation method of performing neuraxial procedures, that relies on the identification of the iliac crests and spinous processes, has a first pass success rate of only 42% in a heterogenous obstetric population with easy or difficult backs.^3^ In this meta-analysis and systematic review, the experience of the operator did not influence the first pass success rate. To the knowledge of the authors, this is the first study to investigate the correlation between the intergluteal cleft and neuraxial midline as determined by ultrasound. In theory, the midline of the spine should be in the same position as the intergluteal cleft. This has been found to be the case with an accuracy of 2 mm or less in approximately 40% and 4.5 mm or less in over 66% of obstetric patients. The supraspinous and interspinous ligament complex connects adjacent spinous processes and has been found to have a median posterior width of 9.2 mm, a middle width of 4.5 mm and an anterior width of 7.3 mm in the lumbar region.^6^ It is hence likely, in our opinion, that the use of the intergluteal cleft may offer some additional benefit in the absence of neuraxial ultrasound when the spinous processes are challenging or impossible to palpate or to reconfirm where the midline of the spine is positioned in situations of difficult neuraxial placement or times of uncertainty.

We acknowledge several limitations of the study. First, it was conducted in an elective manner on obstetric patients who were in the sitting position and not in labour. The findings can therefore be extrapolated to women scheduled for elective caesarean delivery who would be able to sit for the neuraxial block, but may not be relevant if they are in a lateral position, or need labour analgesia or require emergency caesarean delivery where positioning might be more difficult. Second, the position of the intergluteal cleft and neuraxial midline was ascertained by a limited number of investigators. Third, the position of the intergluteal cleft was determined in a nonsterile way with a spirit ruler and does thus not reflect how it would be identified in the clinical environment. Fourth, the position of the neuraxial midline was ascertained by ultrasound, but its accuracy in determining this has never been compared with computed tomography or magnetic resonance imaging. Fifth, the results should not be translated to obstetric patients who have a past medical history of scoliosis or previous spinal surgery or those with a gestation under 37 weeks.

In conclusion, with pregnant women of a gestation ≥37 weeks in the sitting position, the intergluteal cleft can delineate the position of the neuraxial midline, as determined by ultrasound, within a distance of 2 mm in almost 40% of women and 3 mm on average, regardless of the BMI.
